# EXPLORING BARRIERS AND FACILITATORS TO PHYSICAL ACTIVITY IN EUROPEAN MIDLIFE AND OLDER ADULT SAMPLES

**DOI:** 10.1093/geroni/igae098.3481

**Published:** 2024-12-31

**Authors:** Tiana Broen, Stephen Aichele, Leilani Feliciano

**Affiliations:** University of Colorado Colorado Springs, Colorado Springs, Colorado, United States; Colorado State University, Fort Collins, Colorado, United States; University of Colorado Colorado Springs, Colorado Springs, Colorado, United States

## Abstract

Physical activity (PA) is an adaptable and accessible health-promoting behavior with wide-ranging benefits (e.g., reduced risk for diabetes, heart disease, and all-cause mortality). However, only 24.3% of U.S. adults meet current recommendations for PA (150 minutes of moderate-intensity PA and 2 days of muscle strengthening activity per week). It is therefore crucial to understand factors that may support increased engagement in PA. The current study used a random forest machine learning analysis (RFA) alongside a conventional parametric methodology (i.e., linear multiple regression) to evaluate the relative importance of 58 risk/protective factors for PA (moderate and strenuous activity) in a population-representative sample of middle-aged and older adults (N = 67,603; 56.1% women; ages 45-105y; 18 countries) from the Survey of Health, Ageing and Retirement in Europe (SHARE Wave 6). For both women and men, top predictors identified through RFA included variables related to socio-demographics (age, sex, education, country), physical and functional health (mobility, self-rated health, grip strength), as well as variables related to mental health (cognitive performance, depressive symptoms) and socio-relational engagement (caring for others outside of the home, social connectedness). Follow-up hierarchical regressions showed that key predictors accounted for 35% of variability in PA, with contributions as follows: socio-demographic (20%), health/mobility (12%), and cognitive and socio-relational (3%). Together, these results indicate that PA is influenced by multiple bio-psycho-social factors and suggest that future interventions to promote PA may benefit by considering such constellations.

